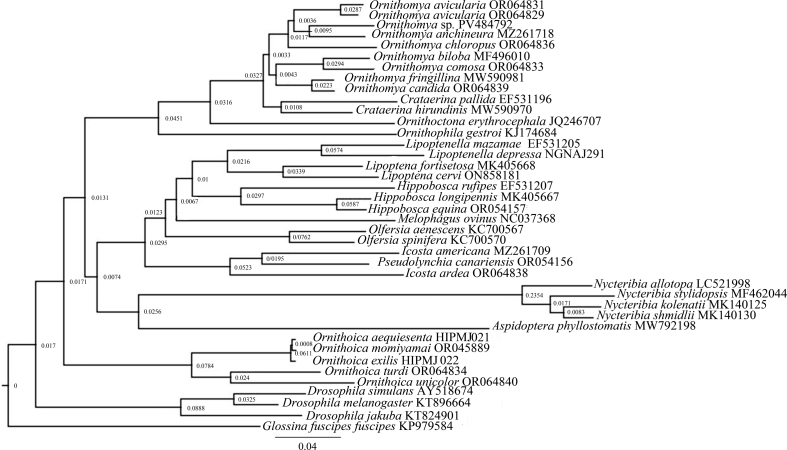# Morphological and genetic variation in the *Ornithomya avicularia* (Diptera: Hippoboscidae) populations across northeastern Eurasia

**DOI:** 10.1016/j.ijppaw.2026.101235

**Published:** 2026-05-06

**Authors:** Tatiana Triseleva, Andrey Safonkin, Alexandr Matyukhin, Anastasia Klueva, Emilia Nartshuk, Mikhail Markovets, Anatoly Shapoval, Valeriy Shokhrin, Sergey Gashkov, Tatiana Rymkevich, Gelena Lunina, Oleg Tolstenkov, Aleksandra Yatsuk

**Affiliations:** a A.N. Severtsov Institute of Ecology and Evolution, Russian Academy of Sciences, 33 Leninskiy Av., 119071, Moscow, Russia; b Federal State Budgetary Institution “Research Institute for Complex Issues of Cardiovascular Diseases”, 6 Academician Barbarash blvd., 650002, Kemerovo, Russia; c Zoological Institute of the Russian Academy of Sciences, Universitetskaya emb. 1, 199034, Saint Petersburg, Russia; d United Administration of the Lasovsky State Nature Reserve Named After L.G. Kaplanov and “Zov Tigra” National Park, 56 Tsentralnaya St., Lazo, Primorskiy Territory, 692980, Russia; e Biological Institute of Tomsk State University, 36 Lenin Av., Tomsk, 634050, Russia; f Nizhne-Svirsky State Nature Reserve, Karla Marksa str. 27/1, 187700, Lodejnoe Pole, Leningrad Region, Russia; g Saint-Petersburg Pasteur Institute, Mira st. 14, 197101, Saint Petersburg, Russia; h Michael Sars Center, University of Bergen, Bergen, Norway

**Keywords:** Louse fly, *Ornithomya avicularia*, Hippoboscidae, Population structure, Russian far east, Eurasia, Europe

## Abstract

Generalist parasites provide valuable systems for examining how broad ecological niches influence patterns of evolutionary diversification. The louse fly *Ornithomya avicularia* (Diptera: Hippoboscidae) is a widespread avian ectoparasite parasitizing a broad range of bird hosts across Eurasia, Australia and Africa. However, the extent to which geographic structure contributes to morphological and genetic differentiation within this species remains poorly resolved. Morphological and molecular variation in *Ornithomya avicularia* was investigated using specimens from seven populations spanning Europe, West Siberia, and the Russian Far East. Morphological variation was largely continuous across most of the studied range, although populations from Siberia and the Russian Far East exhibited consistent differences in wing microtrichial patterns and wing vein ratios. Genetic analyses identified three major haplotype groups, including a widespread European-West Siberian cluster and two deeply divergent lineages restricted to the Russian Far East. One Far Eastern lineage showed genetic distances approaching interspecific levels and was strongly separated in multivariate morphospace, consistent with its recognition as a distinct species described elsewhere. Isolation-by-distance analyses revealed a significant association between geographic distance and morphological differentiation, whereas genetic differentiation showed weaker geographic structuring.

## Highlights

• Integrative analysis reveals geographic structure in *Ornithomya avicularia*.
• A deeply divergent Far Eastern lineage indicates species-level differentiation.
• Western and Siberian populations exhibit a weak morphological cline.

## Graphical abstract

**Figure:**
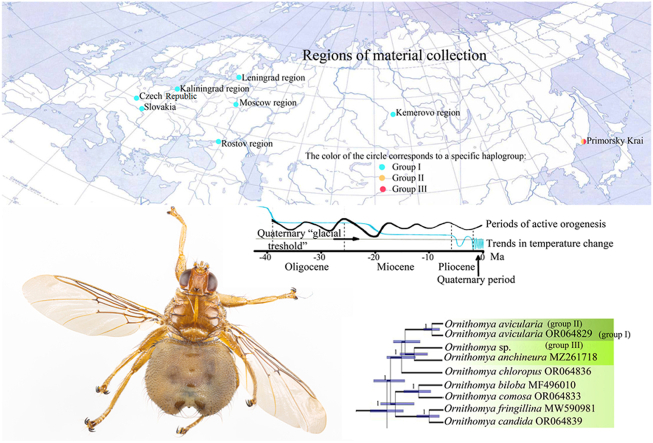

## 1 Introduction

Parasites constitute a major component of global biodiversity and play important roles in host evolution and ecosystem functioning, making them powerful models for investigating how biological diversity is generated and maintained (Chesson, 2000; Parmesan, 2006; Poulin et al., 2011; Jeltsch et al., 2013). Generalist parasites are particularly informative in this context because their broad ecological niches and complex host-parasite networks can both facilitate and constrain evolutionary diversification (Johnson et al., 2009; Martinů et al., 2015). Interpreting evolutionary processes such as adaptation or speciation requires placing observed patterns within explicit temporal and spatial frameworks, which has become increasingly feasible through the widespread use of molecular markers. In insects, integrative approaches combining morphological and genetic data, commonly including cytochrome *c* oxidase subunit I (COI), have proven effective for reconstructing divergence processes, delimiting closely related taxa, and assessing population structure across spatial scales (Whinnett et al., 2005; Johnson et al., 2009; Sychra et al., 2021; Safonkin et al., 2024; Juvé et al., 2025). Resolving the structure of parasite populations and their host-parasite networks is also essential for understanding and predicting the spread of vector-borne diseases (Graham et al., 2009).

Parasitic louse flies (Hippoboscidae Samouelle, 1819) are obligate blood-feeding ectoparasites of birds and mammals (Hutson, 1984) and are recognized as vectors of a wide range of pathogens (Doszhanov, 1980; Farajollahi et al., 2005; Khametova et al., 2018; Santolíková et al., 2022; Peña-Espinoza et al., 2023; Wawman, 2023). In addition, they frequently engage in phoretic associations with mites (Hill et al., 1967; Philips and Fain, 1991) and feather lice (de Moya, 2019; Lee et al., 2022), contributing to complex multi-parasite assemblages. Within Hippoboscidae, the genus *Ornithomya* Latreille, 1802 currently comprises 34 extant and one fossil species (Maa, 1966; Dick, 2006; Yatsuk et al., 2025b), distributed primarily across the Palearctic region. Host specificity within the family varies widely, ranging from narrowly specialized species restricted to a single host group to broad generalists parasitizing numerous bird species and even multiple avian orders (Lehikoinen et al., 2021; Keve et al., 2024).

The most widespread species of the genus, *Ornithomya avicularia* Linnaeus, 1758, exemplifies this generalist strategy. It parasitizes birds from at least 11 avian orders (Maa, 1969; Doszhanov, 2003; Bartos et al., 2020; Lehikoinen et al., 2021; Nartshuk et al., 2022; Santolíková et al., 2022; Mlynárová et al., 2025; Wawman, 2025), although it most frequently exploits passerines and species whose wintering grounds lie relatively close to their breeding areas (Santolíková et al., 2022). Its geographic distribution spans Australia, Africa, Europe, Central Asia and Russia, extending eastwards to Korea and Japan (Doszhanov, 2003; Sychra et al., 2020; Oslejskova et al., 2020). In Europe and Fennoscandia, *O. avicularia* has been described as a “promiscuous specialist”, occurring on numerous passerine species while remaining largely confined to this host clade (Lehikoinen et al., 2021).

A subspecies, *O. avicularia aobatonis* Matsumura, 1905, has been reported from Korea, Japan and probably eastern Siberia (Maa, 1969). In Russia, *O. avicularia* is recorded from the European part, West Siberia and the Far East (Doszhanov, 2003), making it a suitable model for examining geographic structure and diversification in a broadly distributed generalist ectoparasite. More broadly, parasites with wide host spectra offer valuable opportunities to explore how ecological niche breadth interacts with mechanisms of speciation and allopatric divergence (Martinů et al., 2015).

Despite its extensive geographic range and broad host use, the extent and nature of morphological and genetic differentiation within *O. avicularia* remain insufficiently resolved. This apparent generalism raises key systematic and evolutionary questions: does the species’ wide distribution primarily reflect phenotypic plasticity and high dispersal ability, or does it conceal geographically structured evolutionary lineages? Are diagnostic morphological characters detectable against the generally conserved external morphology typical of Hippoboscidae, and do these coincide with patterns of genetic divergence? Addressing these questions is important not only for clarifying the systematic status of *O. avicularia* sensu lato, but also for understanding whether widespread generalist vectors harbor cryptic diversity with potential consequences for parasite transmission dynamics and host-parasite network structure (Graham et al., 2009; Santolíková et al., 2022).

Here, we combine analyses of wing morphology (including wing length, venation ratios, and microtrichial patterns) and scutellar setae counts with COI sequence data from seven *O. avicularia* populations spanning Europe, West Siberia, and the Russian Far East. Using multivariate and distance-based approaches, we evaluate the consistency between morphological and genetic variation, test for isolation by distance, and genetic and phenotypic heterogeneity of Far Eastern populations relative to western and Siberian populations. This integrative framework allows us to clarify patterns of variation within *O. avicularia* across northeastern Eurasia and to explore how broad host use and large geographic ranges shape diversification in generalist parasitic flies.

## 2 Material and methods

### 2.1 Sampling

Specimens of *Ornithomya avicularia* Linnaeus, 1758 were collected between 2017 and 2024 from six localities across Russia and Europe: Kaliningrad Region (Curonian Spit; K), Leningrad Region (Nizhnesvirsky Nature Reserve; L), Moscow Region (Klementyevo village; M), Rostov Region (Rostov-on-Don; R), Kemerovo Region (Kemerovo; Km), and Primorsky Krai (Lazovsky Nature Reserve; P) (Table 1, S1). Material was collected by S. Gashkov, A. Klueva, G. Lunina, M. Markovets, A. Matyukhin, T. Rymkevich, A. Shapoval, V. Shokhrin, O. Tolstenkov, A. Zabashta and M. Zabashta (see Zabashta and Zabashta, 2023).

**Table 1:** Haplotype diversity of *Ornithomya avicularia* populations including *Ornithomya* sp.

Group number	Haplotype number	Region of collection	NCBI number
Group I	H1	Kaliningrad (Curonian Spit)	PV466816
		Kemerovo (Kemerovo)	PV467698
		Slovakia	OP035933
		England	PQ066336 PQ066337
		Poland	OQ029449 OQ029459 OQ029460 OQ029466 OQ029468
	H2	Kemerovo (Kemerovo)	PV467364
		Poland	OQ029463
	H3	Kemerovo (Kemerovo)	PV467370
	H4	Kemerovo (Kemerovo)	PV467375
	H5	Kaliningrad (Curonian Spit)	PV467377
	H6	Kaliningrad (Curonian Spit)	PV467404
	H7	Kaliningrad (Curonian Spit)	PV467418
	H8	Moscow (Klementyevo village)	PV467458
		Czech Republic	MF495992
	H9	Kaliningrad (Curonian Spit)	PV467593
	H10	Moscow (Klementyevo village)	OR064830
		Poland	OQ029455
	H11	Kaliningrad (Curonian Spit)	PV467594
	H12	Kaliningrad (Curonian Spit)	PV467603
	H13	Kaliningrad (Curonian Spit)	PV467707
		Rostov (Rostov on Don)	PV468193
		Poland	OQ029445 OQ029450 OQ029451
	H14	Rostov (Rostov on Don)	OR064829
		Finland	MW590971
	H15	Leningrad (Nizhnesvirsky Nature Reserve)	PV467719
	H16	Leningrad (Nizhnesvirsky Nature Reserve)	PV467726
	H17	Kaliningrad (Curonian Spit)	OR064832
		Poland	OQ029452 OQ029465 OQ029467
	H18	Kaliningrad (Curonian Spit)	PV474178
		Poland	OQ029447
	H19	Kaliningrad (Curonian Spit)	PV474180
	H30	Poland	OQ029464
	H31	Poland	OQ029448
	H32	Finland	MW590975
	H33	England	PQ066332
	H34	England	PQ066333
	H35	England	PQ066334
	H36	Poland	OQ029453
	H37	Poland	OQ029458
Group II	H20	Primorsky Krai (Lazovsky Nature Reserve)	PV474206
	H21	Primorsky Krai (Lazovsky Nature Reserve)	PV474208
	H22	Primorsky Krai (Lazovsky Nature Reserve)	PV474212
	H23	Primorsky Krai (Lazovsky Nature Reserve)	OR064831
	H24	Primorsky Krai (Lazovsky Nature Reserve)	PV474219
	H25	Primorsky Krai (Lazovsky Nature Reserve)	PV474221
	H38	Japan	LC833957 LC833958 LC833961 LC833970 LC833999 LC834009
	H39	Japan	LC833959 LC833960 LC833993 LC834003
	H40	Japan	LC833962
	H41	Japan	LC833963
	H42	Japan	LC833964
	H43	Japan	LC833965 LC833979 LC833985 LC833996 LC834000
	H44	Japan	LC833968
	H45	Japan	LC833969
	H46	Japan	LC833971
	H47	Japan	LC833972
	H48	Japan	LC833973 LC834002
	H49	Japan	LC833981
	H50	Japan	LC833982
	H51	Japan	LC833983
	H52	Japan	LC833986
	H53	Japan	LC833987 LC833988 LC833991 LC833992 LC833997
	H54	Japan	LC833989 LC833998 LC834004
	H55	Japan	LC833990
	H56	Japan	LC833994
	H57	Japan	LC833995
	H58	Japan	LC834001
	H59	Japan	LC834010
Group III	H26	Primorsky Krai (Lazovsky Nature Reserve)	PV478787
	H27	Primorsky Krai (Lazovsky Nature Reserve)	PV484793
	H28	Primorsky Krai (Lazovsky Nature Reserve)	PV484792
	H29	Primorsky Krai (Lazovsky Nature Reserve)	PV484794

**Table 2:** Molecular diversity of the *O. avicularia* populations, calculated in ARLEQUIN v3.5.

Population number, Region^a^	1 M	2 R	3 Slovakia, Czech Republic^b^	4 K	5 P	6 Km	7 L
Number of individuals	2	2	2	14	6	10	2
Total number of haplotypes/unique	2/2	2/2	2/2	11/9	6/6	4/2	2/2
Number of variable sites	3	2	1	17	8	3	4
Transitions/transversions	3/1	2/0	1/0	15/3	8/0	3/0	4/0
(H)	1.0 ± 0.5	1.0 ± 0.5	1.0 ± 0.5	0.96± 0.04	1.0 ± 0.09	0.71± 0.12	1.0 ± 0.05
(π)	0.005± 0.005	0.003± 0.004	0.002± 0.002	0.007 ± 0.004	0.005± 0.003	0.002 ±0.001	0.006 ± 0.007
pi	3.0 ± 2.45	2.0 ± 1.73	1.0 ± 1.0	4.43 ± 2.32	2.87 ± 1.75	0.95 ± 0.71	4.0 ± 3.17

a Designation of collection points, see material and methodology.

b Data from NCBI.

**Table 3:** FST index of *O. avicularia* populations, calculated in ARLEQUIN v3.5.

Population number, Region^a^	1 M	2 R	3 Slovakia, Czech Republic	4 K	5 P	6 Km	7 L
1	0.00000						
2	0.16667	0.00000					
3	−0.14286	0.00000	0.00000				
4	0.02272	−0.23425	−0.12527	0.00000			
5	0.85662	0.83784	0.86497	0.78498	0.00000		
6	0.50257	0.29894	0.19463	0.16606	0.90708	0.00000	
7	0.12500	−0.20000	0.00000	−0.05804	0.83033	0.41450	0.00000

a Designation of collection points, see material and methodology.

Flies were obtained as bycatch during bird ringing at regional ornithological stations and preserved in 96% ethanol. In total, 123 individuals were analyzed genetically, of which 33 were examined morphologically. All specimens are deposited in the working collection of the A.N. Severtsov Institute of Ecology and Evolution, Russian Academy of Sciences (IPEE RAS).

### 2.2 Morphological analysis

Morphological analyses were conducted on 130 individuals. Characters commonly used in the identification of *O. avicularia* were examined, including wing length, number of long scutellar setae, the ratio of costal vein sections (R_1_–R_2+3_/R_2+3_–R_4+5_), and wing microtrichial patterns (Table 4). Additional diagnostic characters were examined to confirm correspondence with *O. avicularia* sensu lato.

**Table 4:** Variability of taxonomic features of *Ornithomya avicularia*.

Feature\Population		K n = 28	M n = 20	R n = 20	L n = 18	Km n = 25	P n = 12	sp. n = 7
Number of large setae on the scutellum	8 (avicularia-type)	24	16	17	14	22	12	7
	6	0	0	0	1	0	0	0
	9	3	3	3	2	3	0	0
	10	1	1	0	1	0	0	0
Wing length. Mean ± standard deviation	-	6.15± 0.29	6.42± 0.40	6.10± 0.27	6.28± 0.39	6.30± 0.40	5.99± 0.11	5.14 ± 0.24
Ratio of sections of costal vein between junctions of R_1_–R_2+3_ and between junctions of R_2+3_–R_4+5_	2:1	25	20	20	18	19	10	7
	1.6:1	2	0	0	0	6	2	0
	2.5:1	1	0	0	0	0	0	0
Arrangement of wing microtrichia	3 stripes in cell 1m, cell 2m bare (avicularia-type)	18	19	20	12	11	12	7
	2 stripes in cell 1 m	5	1	0	3	7	0	0
	1 stripe in cell 1 m	5	0	0	3	7	0	0
	1 stripe in cell 2m	0	0	0	0	0	0	2
Arrangement of wing microtrichia in cell 3r	anchineuric type	0	0	0	0	0	4	3
	transitional type	0	0	0	0	0	4	2

n - Number of flies.

Microtrichial arrangements in wing cells 3r, 1m, and 2m were assessed using published reference figures (Maa, 1963; Doszhanov 1980). Three microtrichial morphotypes were recognized in cell 3r: (i) an avicularia-type, characterized by near-complete coverage except for the basal corner and narrow vein-adjacent stripes (a typical arrangement for this species); (ii) an anchineuria-type, with uniform microtrichial coverage and no central bare area, corresponding to *O. anchineuria* Speiser, 1905; and (iii) a transitional morphotype with a reduced central bare patch displaced toward vein R_4+5_.

Morphological measurements were compared using Student's t-tests implemented in Statistica 8 (StatSoft Inc., USA).

### 2.3 DNA extraction, amplification, and sequencing

Genomic DNA was extracted from whole specimens using Diatom-200 reagents (Isogen, Moscow) following the manufacturer's protocol. A fragment of the mitochondrial cytochrome *c* oxidase subunit I (COI) gene was amplified using primers LCO1490 and HCO2198. Polymerase chain reaction conditions consisted of an initial denaturation at 94 °C for 1 min; six cycles of 94 °C for 1 min, 45 °C for 1 min and 72 °C for 1 min; followed by 40 cycles of 94 °C for 1 min, 55 °C for 1.5 min and 72 °C for 1.5 min; with a final extension at 72 °C for 6 min.

Amplification products were purified by ethanol precipitation using 5 M sodium acetate. Sequencing was performed on an ABI PRISM 3130 automated sequencer (Applied Biosystems, USA) using the BigDye Terminator v3.1 kit. Laboratory work was conducted at the Joint Usage Center “Instrumental Methods in Ecology” (IEE RAS).

### 2.4 Sequence data and phylogenetic analyses

The analyzed COI fragment comprised 637 base pairs for the present morphological dataset and 626 base pairs for the global *O. avicularia* dataset. In total, 99 COI sequences of *O. avicularia* were analyzed (Table 1). Newly obtained haplotypes were deposited in the NCBI database; four previously published sequences were retrieved from GenBank under accession numbers OR06429–OR06432.

Sequence processing and analyses were conducted using the following programs: BLAST (NCBI; https://blast.ncbi.nlm.nih.gov/Blast.cgi) to assess similarity between the obtained nucleotide sequences and those available in the NCBI database; Network v4.6.1.1 (Bandelt et al., 1999) to visualize relationships among haplotypes; MEGA 11 (Tamura et al., 2021) to calculate p-distances; ARLEQUIN v3.5 (Excoffier and Lischer, 2010) to estimate molecular diversity; and BEAST v2.7.7 (Bouckaert et al., 2014) to construct a chronogram. Divergence time estimates were inferred using a relaxed log-normal molecular clock and a Yule speciation model (Yule, 1925). To refine divergence time estimates, two haplotypes from population P were incorporated into a previously published Hippoboscidae chronogram (Yatsuk et al., 2025a). Calibration points included the divergence between *Drosophila melanogaster* and *D. simulans* at 5.1 million years ago (Tamura et al., 2004) and the estimated split between Hippoboscidae and Glossina Wiedemann, 1830 at 34 million years ago (Misof et al., 2014).

### 2.5 Multivariate and statistical analyses

Raw morphological measurements were imported into Python and assembled into a population-by-trait matrix using pandas. Missing values were imputed using column means, and all variables were standardized to a zero mean and unit variance. Morphological distances were calculated as Euclidean distances.

COI sequences were aligned using MAFFT and processed in Biopython. Pairwise uncorrected p-distances (gaps ignored) were calculated and averaged to produce a population-by-population genetic distance matrix.

Principal Coordinates Analysis (PCoA) was applied to both morphological and genetic distance matrices. Congruence between morphological and genetic patterns was assessed using Procrustes analysis based on population coordinates, as well as Mantel tests with 9999 permutations. Geographic distances between populations were calculated from latitude-longitude coordinates using the Haversine formula. Pearson correlation analyses were used to evaluate isolation-by-distance patterns and associations among morphological, genetic, and geographic distances.

All analyses were repeated after exclusion of the Far Eastern lineage (*Ornithomya sp.*, = newly described species *O. panovi* Yatsuk et al., 2025b) to assess robustness. Statistical analyses were conducted in Python 3.11 using pandas, SciPy, scikit-bio, Biopython, and Matplotlib.

## 3 Results

### 3.1 Morphological variation

Most examined specimens of *O. avicularia* (84%) possessed eight large black scutellar setae (Table 4). Variation in scutellar setae number was observed across populations. One specimen from the Kemerovo population (Km) exhibited six large dark setae and two large light setae located at the scutellar margins (Table 4). A single specimen from the Leningrad population (L) possessed only six large scutellar setae (Table 4). Nine scutellar setae were recorded in 10.7-15% of individuals from all populations except Primorsky Krai (P) (Table 4). Ten scutellar setae were present in 3.6% of specimens from Kaliningrad (K), 5.0% from Moscow (M), and 5.5% from Leningrad (L) (Table 4). No statistically significant differences among populations were detected for scutellar setae number. Representative specimens showing variation in scutellar setae are illustrated in Fig. S1A.

Mean wing length across all examined individuals was 6.22 ± 0.35 mm (range 5.7-7.0 mm). The shortest wings were recorded in specimens from population P, whereas the longest wings were observed in population M. Pairwise comparisons using Student's t-tests revealed significant differences in wing length between M and P (*t* = −3.60, *df* = 30, *p* = 0.001), M and K (t = −2.69, *df* = 46, *p* = 0.009), M and R (*t* = −2.96, *df* = 38, *p* = 0.005), L and P (*t* = −2.52, *df* = 28, *p* = 0.017), and Km and P (*t* = −2.65, *df* = 35, *p* = 0.011).

The ratio of the costal vein sections between the junctions R_1_–R_2+3_ and R_2+3_–R_4+5_ was 2:1 in the majority of specimens. This ratio was observed in 89% of individuals from K, 72% from Km, 83% from P, and in 100% of specimens from L, R, and M. Deviations from this proportion did not form consistent population-specific patterns.

The avicularia-type arrangement of wing microtrichia in cell 3r was present in 93.5% of examined individuals. Deviations from this pattern were restricted to eight individuals from population P. In four of these specimens, the microtrichial pattern corresponded to that described for *O. anchineuria* Speiser, 1905, characterised by uniform microtrichial coverage without a central bare area. The remaining four specimens exhibited a transitional morphotype, with a reduced central bare area displaced toward vein R_4+5_. No individuals from populations K, L, M, R or Km showed deviations from the avicularia-type pattern in cell 3r (Fig. S1B).

The avicularia-type microtrichial arrangement in cell 1m was present in 75% of individuals overall. In populations K, Km and L, some specimens exhibited a single microtrichial stripe in cell 1m, whereas in K, Km, L and M, two microtrichial stripes were occasionally observed (Fig. S1). The proportion of individuals retaining the avicularia-type pattern was lowest in population Km (48%), compared with 64% in K, 67% in L, 95% in M, and 100% in both R and P.

Examination of material from the Far Eastern region revealed a subset of specimens that differed markedly from typical *O. avicularia* in several characters. These individuals showed differences in wing length, the ratio of costal vein sections (R_1_–R_2+3_/R_2+3_–R_4+5_), and additional morphological characters, including strong reduction of tergite 3. Some possessed a microtrichial stripe in cell 2m, a character typical of *O. anchineuria*. These specimens were distinct from individuals from the European part of Russia and southern Siberia and from other individuals within population P. These specimens are herein referred to as *Ornithomya* sp.; their formal description is provided elsewhere (Yatsuk et al., 2025b).

### 3.2 Molecular diversity

Median-joining haplotype network analysis based on COI sequences revealed three distinct haplotype groups (Fig. 1; Supplementary Fig. 2). Group I comprised all European and Siberian populations (K, M, R, L and Km), as well as reference sequences from England, Poland, Finland, Slovenia and the Czech Republic. Groups II and III were restricted to the Far Eastern population (P) and reference sequences from Japan.

**Fig. 1:**
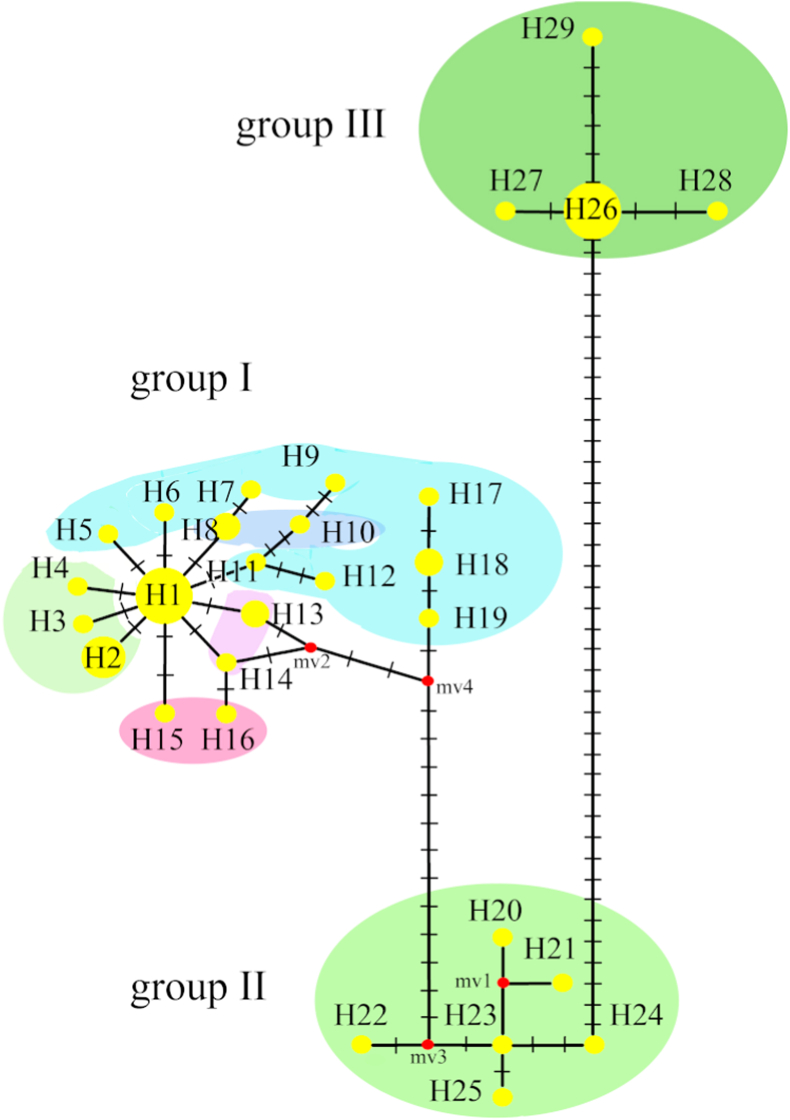
Median haplotype network (made in Network v4.6.1.1) of samples of *Ornithomya avicularia* from different regions based on the nucleotide sequence of the mtDNA COI gene. Yellow circles – individual haplotypes; the diameter corresponds to the number of samples. The red dot marks the ancestral haplotype; lines on branches – mutated positions; multicolored clouds – genetic groups. The sequences included in the H-haplotypes are shown in Table 1.

Genetic distances between Groups I and II ranged from 2.4% to 3.1% (Table 5). Genetic distances among populations within Group I ranged from 0.1% to 0.7%. Group III was the most divergent, with genetic distances of 6.6-7.5% relative to both Groups I and II.

**Table 5:** Genetic distances, calculated in MEGA 11, between all known *Ornithomya avicularia* geographical groups (%).

Place	M	K	sp.	P	R	Km	L	Japan	England	Poland	Slovakia	Finland
K	0.6											
sp.	7.6	7.4										
P	2.9	2.7	6.6									
R	0.5	0.5	7.2	2.4								
Km	0.4	0.5	7.4	2.6	0.3							
L	0.6	0.6	7.4	2.6	0.4	0.4						
Japan	3.1	2.8	6.6	0.5	2.6	2.8	2.7					
England	0.8	0.7	7.1	2.5	0.5	0.6	0.6	2.7				
Poland	0.7	0.6	7.2	2.6	0.4	0.5	0.6	2.7	0.6			
Slovakia	0.3	0.4	7.3	2.6	0.2	0.1	0.3	2.7	0.5	0.4		
Finland	0.6	0.6	7.1	2.4	0.2	0.4	0.4	2.5	0.5	0.5	0.2	
Czech Republic	0.2	0.5	7.5	2.8	0.3	0.3	0.5	2.9	0.7	0.5	0.2	0.4

Pairwise comparisons between populations with available morphological data and reference sequences from Slovenia and the Czech Republic are presented in Table 2, Table 3. FST values indicated high genetic differentiation between population P and all other populations, and moderate differentiation between population Km and the remaining populations.

Alignment of 38 COI sequences from individuals belonging to Groups I and II revealed 39 variable sites, of which 24 were parsimony-informative. A total of 25 haplotypes were identified, 20 of which were unique. Haplotype frequencies ranged from 0.026 to 0.184. The dataset contained 49 transitions and 20 transversions.

Chronogram analyses estimated the initial split within the *O. avicularia* lineage at approximately 7.7 million years ago. Divergence between Groups I and II was dated to approximately 2.2 million years ago, whereas separation of Group III occurred at approximately 6.0 million years ago (Fig. 2, Fig. S5).

**Fig. 2:**
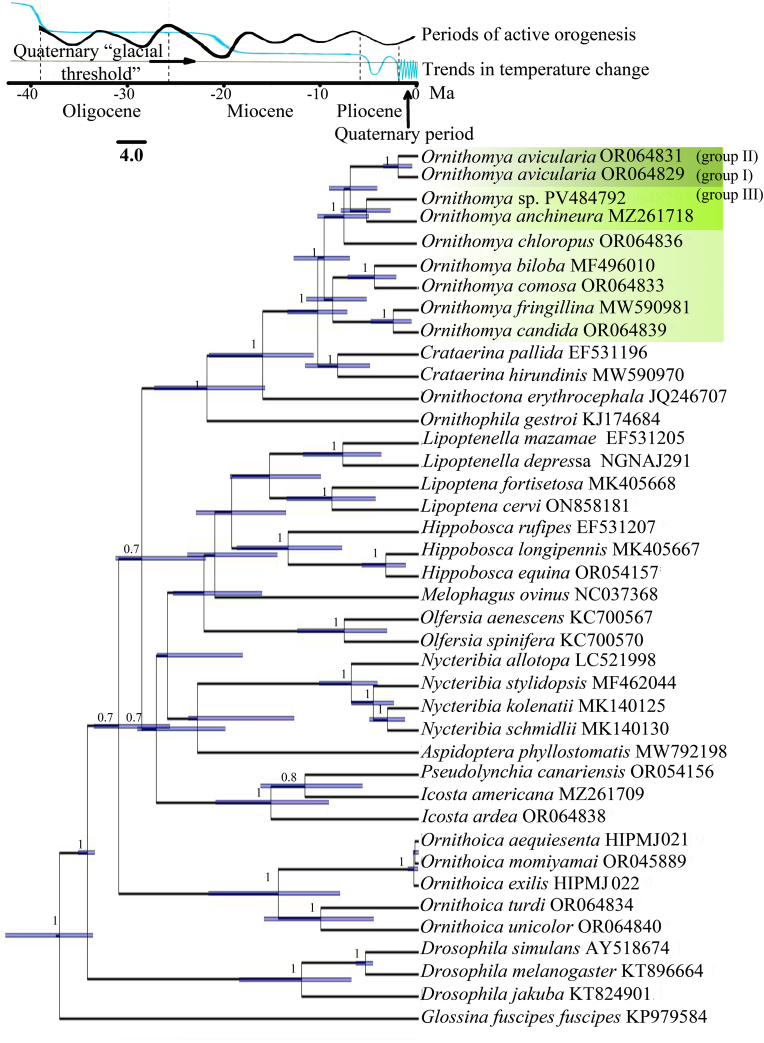
Chronogram of speciation of flies of the family Hippoboscidae with the temperature and mountain-building processes in the Neogene and Quaternary periods, inferred from the dataset CO1 mtDNA using BEAST ver. 2.7.7. Bootstrap support is depicted at the nodes. The purple lines indicate the time period when the groups split. The scale bar depicts divergence estimates in millions of years (Ma). Mountain-building activity line modified from Brian (1979). Lines of trend in temperature change in the Neogen and Quaternary periods, and Quaternary Glacial threshold modified from Klige et al. (1998). The studied genus is highlighted in green. The scale bar depicts divergence estimates in millions of years (Ma).

### 3.3 Morphological-genetic congruence

Patterns of morphological and genetic differentiation were explored using ordination and distance-based approaches (Fig. 3; Figs. S3 and S4). Principal Coordinates Analysis (PCoA) of morphological distances revealed pronounced spatial structure among populations (Fig. 3A). The Far Eastern lineage (*Ornithomya* sp.) was clearly separated along PC1, while the Kemerovo population (Km) was displaced primarily along PC2. In contrast, European populations clustered more closely together, showing partial overlap in morphospace.

**Fig. 3:**
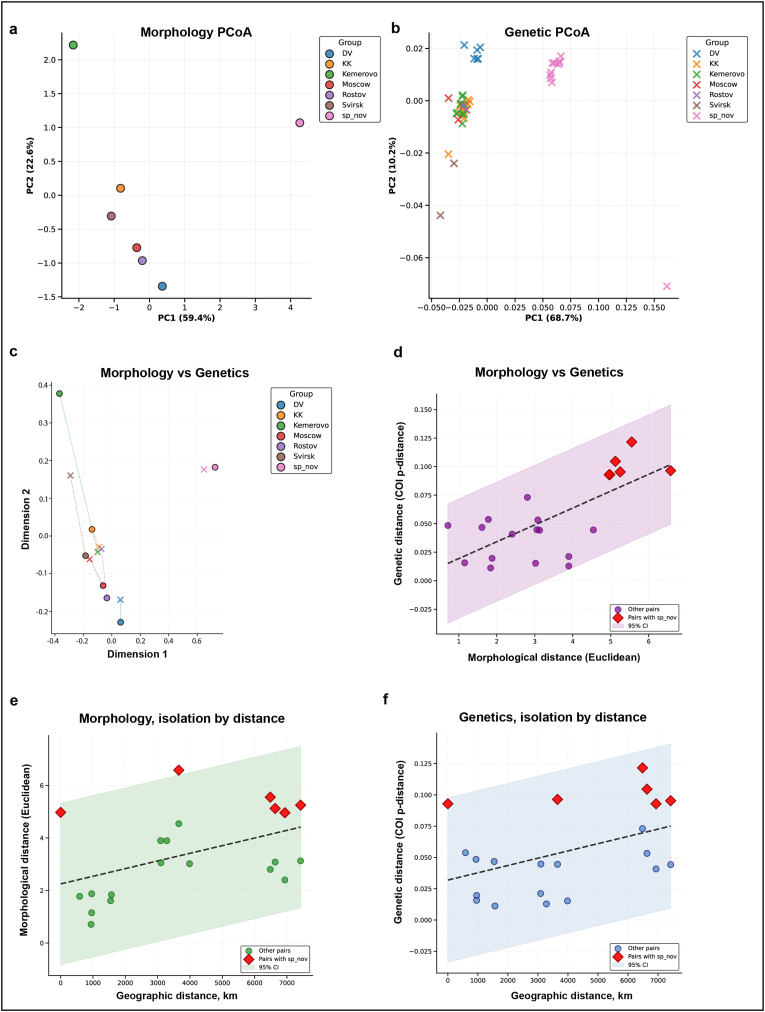
Morphological-genetic congruence and geographic distance effects in *Ornithomya avicularia*. A – Principal Coordinates Analysis (PCoA) of morphological distances for all populations, based on Euclidean distances calculated from standardized morphometric traits. B – PCoA of COI genetic distances for all populations based on uncorrected p-distances. C – Procrustes superimposition of morphological (circles) and genetic (crosses) ordinations for all populations; dashed lines connect corresponding population points (Procrustes disparity = 0.3520). D – Relationship between pairwise morphological and genetic distances for all populations (Pearson r = 0.469, p = 0.0334, slope = 0.0148, n = 21); red diamonds indicate comparisons involving the Far Eastern lineage (*Ornithomya* sp.). E − Relationship between geographic distance and morphological distance (Pearson r = 0.469, p = 0.0334, slope = 0.0003, n = 21). F – Relationship between geographic distance and genetic distance (Pearson r = 0.445, p = 0.0437, slope ≈ 0.0000, n = 21). Dashed lines indicate linear regression fits; shaded areas represent 95% confidence intervals.

PCoA of COI genetic distances showed a different pattern (Fig. 3B). Although the first axis explained a comparable proportion of variance (68.7%), European and Siberian populations largely overlapped in genetic space. Only the Far Eastern lineage formed a clearly isolated genetic cluster, indicating strong mitochondrial differentiation relative to all other populations.

Direct comparison of morphological and genetic ordinations using Procrustes analysis revealed moderate incongruence between the two datasets (*Procrustes disparity* = 0.352; Fig. 3C). Permutation testing indicated that this alignment was marginally non-significant (*p* = 0.067), suggesting that morphological and genetic differentiation are not tightly coupled across the full dataset.

This pattern was further supported by pairwise distance comparisons (Fig. 3D; Fig. S4). When all populations were included, morphological and genetic distances were significantly correlated (*Pearson r* = 0.469, *p* = 0.033). However, this relationship was driven largely by comparisons involving the Far Eastern lineage. After exclusion of this lineage, the correlation disappeared entirely (*Pearson r* = −0.023, *p* = 0.935; Fig. S3D), indicating that, among the remaining populations, morphological differentiation is not mirrored by mitochondrial divergence.

### 3.4 Geographic distance effects

Geographic distance was significantly associated with morphological differentiation when all populations were considered (*Pearson r* = 0.469, *p* = 0.033; Fig. 3E). Importantly, this relationship remained significant after exclusion of the Far Eastern lineage (*Pearson r* = 0.520, *p* = 0.045; Fig. S3E), indicating a consistent isolation-by-distance pattern for morphology across the European and Siberian range.

In contrast, genetic distances showed weaker geographic structuring. When all populations were included, genetic distance increased significantly with geographic distance (*Pearson r* = 0.445, *p* = 0.044; Fig. 3F). However, this pattern was no longer significant after removal of the Far Eastern lineage (*Pearson r* = 0.390, *p* = 0.153; Fig. S3F, Fig. S4), indicating limited isolation by distance in mitochondrial variation among the remaining populations.

Together, these analyses show that morphological variation exhibits a gradual, geography-dependent pattern across the western part of the species’ range, whereas mitochondrial genetic differentiation is dominated by the presence of a deeply divergent Far Eastern lineage.

## 4 Discussion

Our integrative analysis reveals two contrasting patterns within *O. avicularia*: largely continuous, geography-dependent variation across Europe and Siberia, and a discrete, morphologically and genetically divergent lineage in the Russian Far East. This combination illustrates how a widespread generalist ectoparasite can simultaneously exhibit clinal population structure and deep allopatric divergence across its range.

### 4.1 Morphological variability and taxonomic features

For most species of *Ornithomya*, wing length varies by no more than 1 mm (Nartshuk et al., 2024). Our data confirm that *O. avicularia* generally falls within the range of 5.7–7.0 mm. However, the shortest wings (<5.7 mm) observed in Primorsky Krai most likely correspond to *Ornithomya* sp. rather than *O. avicularia* sensu stricto, consistent with earlier observations on Far Eastern material (Doszhanov, 2003; Yatsuk et al., 2025b). Taken together, published data and our measurements suggest that the typical wing length of *O. avicularia* sensu stricto is close to 6.0 mm and clearly exceeds 5.5 mm.

The number of long apical scutellar setae is most often cited as eight in *O. avicularia*, although values ranging from six to ten have been reported. Six setae occur in *O. avicularia aobatonis* from Japan, currently regarded as a synonym of *O. avicularia* (Mogi et al., 2023), whereas counts of up to ten were noted by Maa (1967). Doszhanov (1980) reported a range of seven to ten setae, while more recent studies mention only eight (Doszhanov, 2003; Oboňa et al., 2022). Our results confirm a broad range of six to ten setae and further show that the outer scutellar setae may be pale, a condition also known from *O. chloropus* (Doszhanov, 1980). These findings indicate that scutellar setae number and coloration should be used cautiously and always in combination with other characters.

The ratio of costal vein sections (R_1_–R_2+3_/R_2+3_–R_4+5_) has traditionally been regarded as stable in *O. avicularia* (Doszhanov, 1980, 2003). Nevertheless, our measurements revealed deviations in some populations, indicating that this character may vary at the population level. Wing microtrichial patterns were generally consistent with the *O. avicularia* typical arrangement (Maa, 1963; Doszhanov, 1980), but notable variation was observed in the Kemerovo and Primorsky Krai populations. In particular, individuals from the Far East displayed anchineuric-type features, including uniform microtrichial coverage of cell 3r, microtrichia in cell 2m, and reduction of tergite 3. These traits are taxonomically informative but also demonstrate a degree of intraspecific variability within *O. avicularia*. Similar variation has also been reported in *O. fringillina*, including cases of overlap with other species of the genus (Petersen et al., 2007a, Petersen et al., 2007b; Wawman et al., 2025). Microtrichial characters in cells 1m and 3r, although widely used in identification keys, should therefore be applied with caution and always in conjunction with additional morphological features.

Within the Far Eastern material, we further detected a subset of specimens that differed consistently in wing length, costal vein ratios, microtrichial patterns (including the presence of a stripe in cell 2m), and other external traits such as a strong reduction of tergite 3. These specimens differed not only from European and West Siberian *O. avicularia*, but also from other individuals within the Primorsky Krai population. The extent and consistency of these differences correspond to species-level divergence and match the lineage described as *Ornithomya* sp. by Yatsuk et al. (2025b).

### 4.2 Genetic structuring in hippoboscidae context

In Diptera, intraspecific COI divergence spans a wide range, from <1% in some blow flies and midges to several percent in certain biting midge and sciaroid lineages (Harvey et al., 2003; Linton et al., 2003; Al-Shami et al., 2009; Jomkumsing et al., 2021; Kjærandsen, 2022). Interspecific distances often overlap with the upper range of intraspecific variation, typically falling between ∼1% and 8% or higher depending on the group (Park et al., 2009; Renaud et al., 2012). Within Hippoboscidae, interspecific COI distances generally exceed 8–8.9% (Petersen et al., 2007a; Yatsuk et al., 2024a), whereas intraspecific distances in *Lipoptena cervi* (Linnaeus, 1758) and *L. fortisetosa* Maa, 1965 range from 0.0 to 1.8% (Yatsuk et al., 2024a). In some genera (e.g., Pseudolynchia), interspecific distances may be lower but still typically exceed 3% (Lee et al., 2025).

Against this background, the ∼2.5% COI divergence separating *O. avicularia* sensu stricto (Group I: Europe and West Siberia) from the Far Eastern lineage (Group II) indicates pronounced population structure but does not necessarily imply species-level separation. Similar divergent haplotypes from the Amur region were reported by Meiβner et al. (2020) as “*Ornithomya* A″, corresponding to our Group II from Primorsky Krai. In contrast, the Far Eastern *Ornithomya* sp. (Group III) differs by 6.6-7.3% from both Groups I and II and shows only 92.15-93.56% sequence similarity to *O. avicularia* in BLAST searches. This level of genetic divergence, together with consistent morphological differentiation, strongly supports recognition of Group III as a distinct species (Yatsuk et al., 2025b).

High FST values between the Primorsky Krai population and all other populations coincide with the distinctive microtrichial patterns observed in cell 3r, whereas the moderate FST separating the Kemerovo population from western populations corresponds to more subtle differences in wing morphology and microtrichial arrangement. At the same time, relatively low nucleotide diversity in the Kemerovo population suggests low internal variability, indicating a comparatively homogeneous population characterized by a consistent set of morphological traits.

Chronogram analyses place the split of the *O. avicularia*+*O. anchineuria* lineage at approximately 7.7 million years ago, the divergence between *O. anchineuria* and *Ornithomya* sp. at about 5.8 million years ago, and the separation of European *O. avicularia* at roughly 2.2 million years ago. These estimates are consistent with previously published results (de Moya, 2019; Yatsuk et al., 2024b).

Major avian lineages are thought to have originated no later than ∼30 million years ago (Prum et al., 2015), making it unlikely that the origin of these louse fly lineages is directly linked to the initial radiation of their avian hosts. Diversification within *O. avicularia* and related taxa more likely reflects later geographic isolation and ecological differentiation acting within an already established host assemblage.

### 4.3 Morphology-genetics congruence and geographic effects

Our results indicate that morphological and genetic differentiation in *O. avicularia* respond differently to geographic structure. Across Europe and Siberia, morphological variation follows a gradual geographic pattern, whereas mitochondrial genetic structure is weak, suggesting that phenotypic differentiation accumulates more readily with spatial separation than neutral genetic divergence.

In contrast, the Far Eastern lineage exhibits concordant morphological and genetic differentiation, consistent with long-term isolation and independent evolutionary history. This contrast highlights that *O. avicularia* comprises both clinally structured populations and discrete evolutionary units, depending on regional history and connectivity. Similar decoupling between morphological and genetic patterns has been reported in other insects and may reflect local adaptation, phenotypic plasticity, or demographic processes acting on different timescales (Martinů et al., 2015).

### 4.4 Evolutionary and biogeographic implications

Range dynamics in parasites are shaped by a combination of intrinsic dispersal capacity and host movements. When suitable conditions arise beyond the current distribution, high dispersal potential can facilitate rapid range expansion (Krause et al., 2016; Dudaniec et al., 2022). While many range shifts are directionally consistent with climate change, they may also depend on additional biotic and abiotic factors, including habitat structure and interspecific interactions (Lawlor et al., 2024). In the boreal zone, adult louse flies are active primarily during the summer months, whereas much of their life cycle is spent in the puparial stage (Doszhanov, 1980).

For *O. avicularia*, a generalist ectoparasite exploiting a wide range of avian hosts, its extensive Eurasian distribution is most plausibly explained by repeated bird-mediated dispersal events, including movements of migratory hosts (Doszhanov, 2003; Keve et al., 2024). This scenario is consistent with the observed combination of high haplotype richness and relatively low nucleotide diversity across European and Siberian populations, a pattern often associated with recent or ongoing range expansion (Abramson, 2007; Diome et al., 2012; Miraldo et al., 2014; Dudaniec et al., 2022).

In contrast, the deeply divergent Far Eastern *Ornithomya* sp. likely reflects long-term persistence in regional refugia during Neogene climatic fluctuations (Popova et al., 2012), allowing substantial genetic and morphological divergence to accumulate (Sparks et al., 2014). Paleoenvironmental reconstructions indicate that intensified mountain building and increasing climatic zonality in Eurasia during the late Miocene and Pliocene (Sinitsyn, 1965; Brian, 1979; Klige et al., 1998; Popova et al., 2012; Trifonov and Sokolov, 2014; Jung and Prange, 2020) created heterogeneous landscapes and potential barriers to dispersal. Our divergence estimates (approximately 2.2-6 million years ago) overlap with these environmental transitions, providing a plausible temporal context for diversification within the *O. avicularia*-*O. anchineuria* complex.

Taken together, these results show that even a broadly distributed, host-generalist louse fly can harbor deep allopatric divergence alongside clinal, geography-driven variation. This combination highlights the importance of integrating morphology, genetics and geography when interpreting diversification patterns in ectoparasite vectors.

## 5 Conclusions

Our study demonstrates that broad ecological generalism does not preclude diversification in Hippoboscidae. *Ornithomya avicularia* sensu stricto exhibits predominantly geography-driven clinal morphological variation across Europe and Siberia, accompanied by weak mitochondrial genetic structure. In contrast, the Far Eastern lineage represents a distinct evolutionary unit that is consistently diagnosable using both morphological and molecular data.

These findings underscore the value of integrative taxonomic approaches in louse flies, particularly where widely used diagnostic characters show substantial intraspecific variability. More broadly, they highlight that widespread generalist ectoparasites may harbor cryptic diversity, with important implications for taxonomy, biogeography and the interpretation of host-parasite associations across large spatial scales.

## CRediT authorship contribution statement

**Tatiana Triseleva:** Writing – review & editing, Writing – original draft, Methodology, Formal analysis, Data curation, Conceptualization. **Andrey Safonkin:** Writing – review & editing, Writing – original draft, Methodology. **Alexandr Matyukhin:** Writing – review & editing, Writing – original draft, Methodology, Conceptualization. **Anastasia Klueva:** Writing – review & editing, Writing – original draft, Methodology. **Emilia Nartshuk:** Writing – review & editing, Writing – original draft, Methodology, Conceptualization. **Mikhail Markovets:** Writing – review & editing, Writing – original draft, Methodology. **Anatoly Shapoval:** Writing – review & editing, Writing – original draft, Methodology. **Valeriy Shokhrin:** Writing – review & editing, Writing – original draft, Methodology. **Sergey Gashkov:** Writing – review & editing, Writing – original draft, Methodology. **Tatiana Rymkevich:** Writing – review & editing, Writing – original draft, Methodology. **Gelena Lunina:** Writing – review & editing, Writing – original draft, Methodology. **Oleg Tolstenkov:** Writing – review & editing, Writing – original draft, Visualization, Methodology, Conceptualization. **Aleksandra Yatsuk:** Writing – review & editing, Writing – original draft, Visualization, Methodology, Formal analysis, Data curation, Conceptualization.

## Statements and declarations

The authors declare no competing interests. This research was not conducted on vertebrates. Furthermore, hippoboscid flies are not a protected species and no ethical approval or research permits were required. All sequence data generated in this study are deposited in GenBank under the accession numbers provided in the manuscript. All other data supporting the findings of this study are included in the article and its supplementary materials.

## Conflict of interest statement

The authors declare no conflicts of interest.

## Figures and Tables

**figs1:**
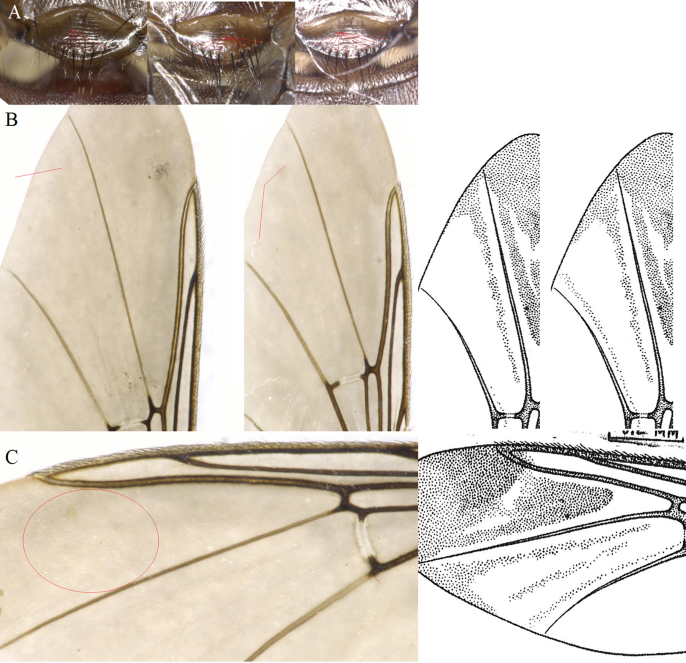


**figs2:**
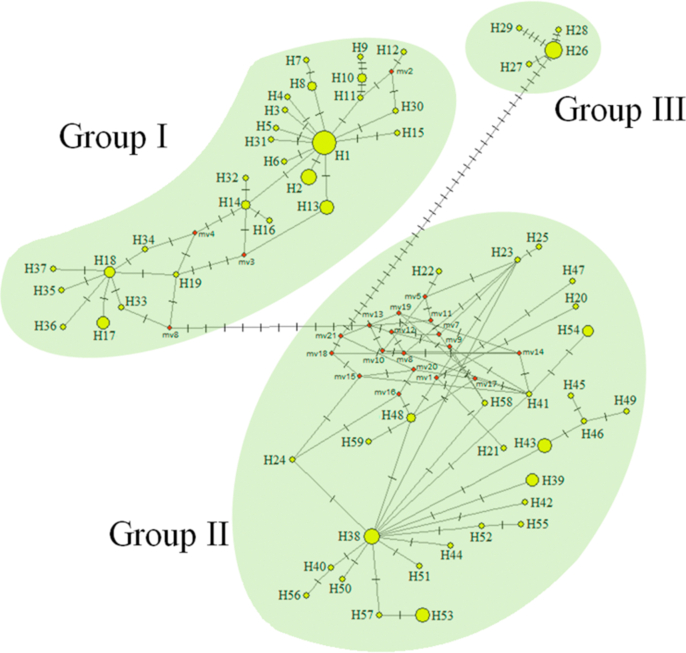


**figs3:**
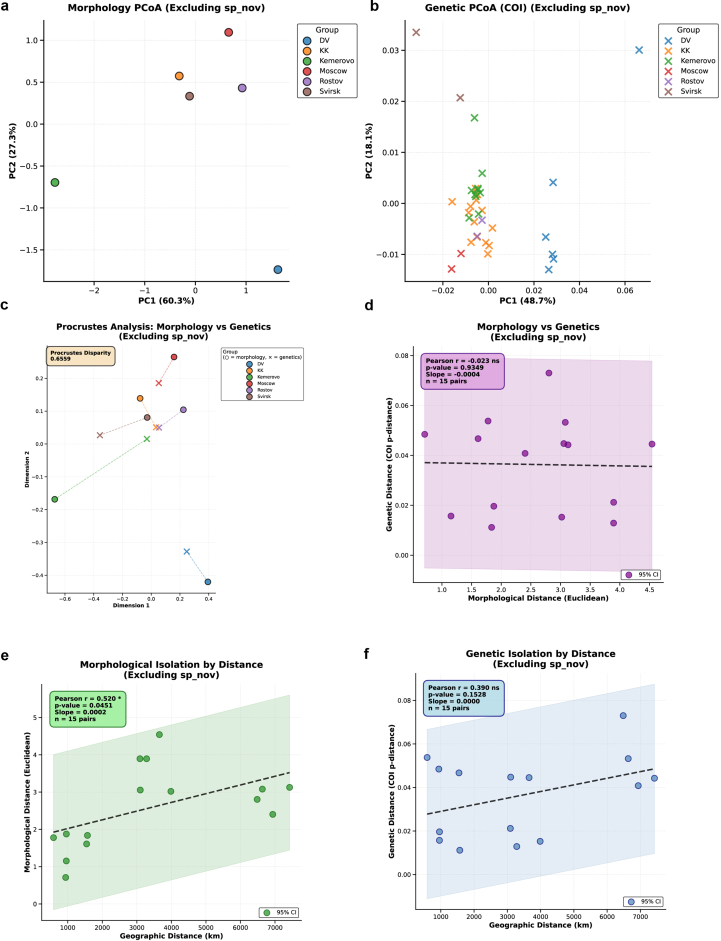


**figs4:**
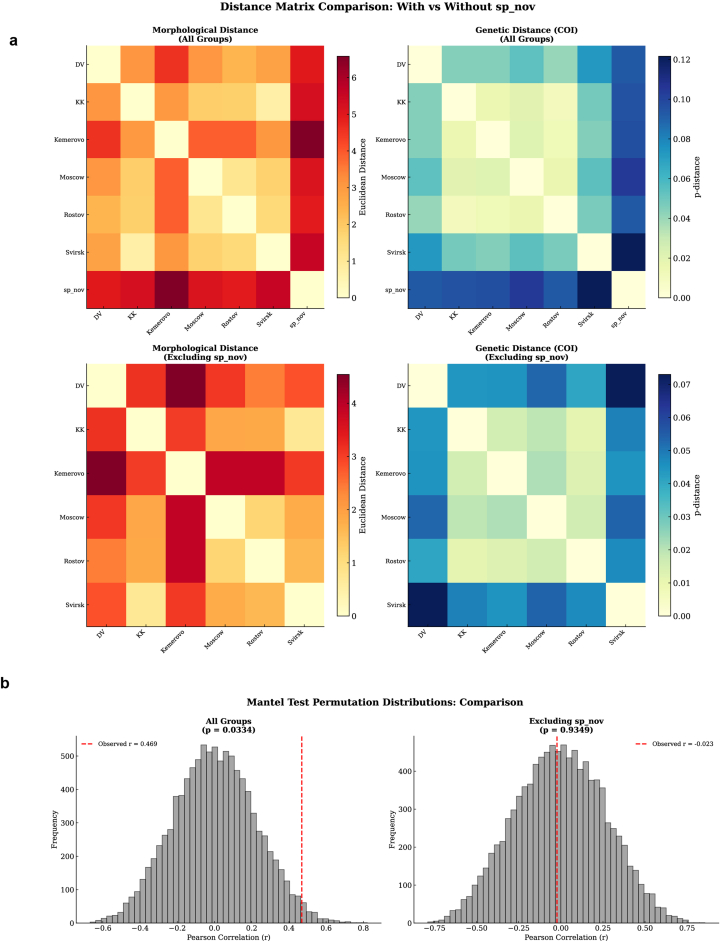


**figs5:**